# Predictors of Health Seeking Behaviours for Common Childhood Illnesses in Poor Resource Settings in Zambia, A Community Cross Sectional Study

**DOI:** 10.3389/fpubh.2021.569569

**Published:** 2021-05-19

**Authors:** Golden Apuleni, Choolwe Jacobs, Patrick Musonda

**Affiliations:** Department of Epidemiology and Biostatistics, School of Public Health, University of Zambia, Lusaka, Zambia

**Keywords:** common childhood illnesses, health seeking behaviours, maternal and child health care, model, poor resource settings

## Abstract

**Background:** Developing countries, including Zambia, account for larger share of child morbidities and mortalities due to common childhood illnesses. Studies on wider determinants of behaviour pertaining to treatment seeking for childhood febrile illnesses in poor resource settings are limited. This study investigated health seeking behaviours of mothers in poor resource settings of Zambia and identified associated factors.

**Methods:** Secondary data from a community cross sectional study design from the Health for the Poorest Population (HPP) Project was analysed between March and May 2019. Data was collected between May and August, 2013. It was collected by means of administering a structured questionnaire from the mothers of under-five children. The survey took place in Samfya and Chiengi of Luapula province while in Northern Province, Luwingu and Mungwi were settled for. A total of 1 653 mothers of under 5 years who had an episode of diarrhoea, malaria, pneumonia or a combination of any of them not more than 14 days before the interview were included in the study. A sample size was arrived at using A Lot Quality Assurance Sampling (LQAS) method. In order to determine the associations between respondent's demographic characteristics and health seeking behaviour, chi square test of independence was carried out. Multivariable logistic regression was also done to identify predictors of health seeking behaviours for common childhood illnesses in children aged <5 years old in poor resource settings.

**Results:** Among the mothers interviewed, 64.6% were married while 35.4% were unmarried. Their mean age was 32 years. Mothers who took their sick children to the health facilities for the purpose of seeking health care for their child for either of the illnesses accounted for 75.2%, [95% CI: 0.62–0.96], while 24.8% did not seek health care for their sick child. Factors typically associated with health seeking behaviours were mothers' marital status [aOR = 0.74; 95% CI: 0.58–0.94], and mothers ‘education level [aOR = 1.47; 95% CI: 1.13–1.92].

**Conclusion:** It was established in this study that health care seeking behaviours for these common childhood illnesses in poor resource settings was relatively high and could be predicted by mother's education level and mothers' marital status. Integrating interventions targeted at increasing utilisation of maternal and child health services with basic education to women and moral support counselling to families may potentially maximise health seeking behaviours in marginalised communities.

## Introduction

Common childhood illnesses remain responsible for a disproportionate number of deaths in under 5 years children in poor resource countries (1). This is despite the global decline in number of under-five deaths from 12.7 million in 1990 to 5.3 million in 2018 (2–4). In spite of recording this success, many countries still record high numbers of under five deaths. Interestingly, these deaths mostly are from preventable or treatable diseases (4). Developing countries account for larger share of these deaths (98.7%) (2), with sub-Saharan Africa being the larger contributor (2, 3). Acute respiratory infections, diarrheal diseases, and malaria which are all preventable and curable, accounted for large proportion of under-five deaths (2–5). Most of these lives could have been saved through readily available treatments such as antibiotics for acute respiratory infections, oral rehydration for diarrheal diseases and antimalarial for malaria (6, 7). In developing countries however, many children's lives continue to be lost due to inappropriate treatment or not seeking health care from health facilities coupled by delays in health care seeking by mothers (6, 8).

In Zambia, mothers' health care seeking behaviour is equally not pleasing, as only a handful of sick under-five years children are attended to at health facilities (9). Nationally, only 27% of under-five children with symptom of acute respiratory infection, 24.2% with fever and 32% with diarrhoea were taken to health facilities during 5 years preceding 2013 (10). It can therefore be concluded that children who were taken to the health facilities for common childhood illnesses are relatively few. Not only that, when health care is sought, it is delayed (11, 12). Home care, visiting traditional healers during illnesses were reported to be a common practise in rural Zambia (9, 10).

Empirical evidence suggests that disease burden and deaths from common childhood illnesses can be reduced considerably if appropriate health care is sought (13). Mothers' ability to recognise and seek appropriate health care is essential in preventing child mortality in developing countries where significant numbers of the children continue to die from childhood febrile illnesses (6, 14). Unfortunately, poor and delays in care seeking for febrile illnesses has been reported in some low income countries (15, 16), including Zambia (9).

A number of studies have been conducted to determine the predictors of health seeking behaviours by mothers for these childhood febrile illnesses, in which different factors have been identified. Among them are structural factors such as distance to the health facilities (17), cultural beliefs, income (6), and mother's livelihoods (16); and socio-demographic determinants namely: age of the mother (18), mother's education level (19–21), mother's marital status (22–24), child's age (22, 23), and sex of the child (7, 25). Few studies however, have been done in poor resource settings to determine health seeking behaviours and associated factors. Evidence therefore, patterning to health seeking behaviours in poor resourced populations is limited. In Zambia, health seeking behaviours for febrile illnesses and associated predictors in poor resourced settings are not well-understood. This study therefore, focused on some economically marginalised populations in order to investigate mothers' health seeking behaviours for common childhood illnesses in children aged <5 years and their determinants in Zambia.

## Materials and Methods

### Study Design

The study was a community based cross sectional study design that used secondary data that were collected under the Health for the Poorest Populations (HPP) Project. Data were collected between May and August, 2013. It was analysed between March and May 2019 using STATA version 15 (STATA corp. college station, Taxes USA).

### The Health for the Poorest Populations Project

The health for the Poorest Populations Project was in response to the high Child and Maternal Mortality ratios in Zambia (26). A number of factors were identified to be contributing to this situation. Among them were: critical shortage of skilled human resource; inadequate funding; inadequate equipment; inadequate essential drugs and supplies; social and cultural barriers to key family practises; and ineffective mechanisms for targeting the poor populations. Detail of the project has been described elsewhere (26).

### Study Setting

Four districts (Mungwi, Luwingu, Samfya, and Chiengi) were identified as some of the districts with most vulnerable and marginalised populations in the country. Their main source of income is through subsistence farming. These districts were selected after analysis of national data on the distribution of vulnerability, poverty, deprivation, and rights failures (27). Northern and Luapula Provinces are among the provinces recording a high number of women dying while giving birth and a lot of children dying before reaching their firth birthday (28). This study focused on populations served by Rural Health Centres. These facilities are manned by frontline health care workers such as Clinical Officers, Nurses, Environmental Health Technologist, and Community Health Assistants.

### Sampling Design and Sample Size

The study was a cross-sectional household survey based on the lot quality assurance sampling (LQAS) method. LQAS was used as guided by the WHO and other related studies (26). Using the LQAS method, a district is considered an independent site, and sub-divided into community clusters regarded as supervisory areas. A supervisory area or community cluster constitutes a catchment area with a dedicated health facility responsible for delivering health services (26, 29). In this study, the four selected districts had a total of 29 community clusters, nine from both Chiengi and Samfya, six from Luwingu, and five from Mungwi. Using the WHO LQAS guide, a list of all the villages in each community cluster was retrieved from the 2010 population census, and the sampling proportionate to size technique was followed to randomly select 19 households from each community cluster (26). A sampling frame was used to select households from which the individual samples were taken. The main criterion for inclusion of households was the presence of mothers with children aged 0–5 months and mothers that lived in the study site during pregnancy and delivered their baby within the same study area. In instances where two or more respondents were found in one household, and met the criteria, random sampling was done to select one respondent. Therefore, a total sample size of 551 participants was used per disease (diarrhoea, malaria, and pneumonia). Detail of sampling for the study has been described elsewhere (26).

### Data Collection

Data collection was done in 2013. It was collected using a questionnaire which was administered by an interviewer. The questionnaire was structured and had questions on mother's background information such as age of the mother, whether mother was married, how far they had gone with their education, when the child experienced any of these symptoms of illness or a combination of any, and whether the mother had taken the child to the health facility during illness. The symptoms that were associated with these common childhood illnesses were: Cough with shortness of breathing for pneumonia; three or more loose or watery stools per day for diarrheal diseases; and body hotness for malaria (30). These were used to determine mothers' health care seeking behaviours.

### Data Quality Control

Research Assistants fluent in local language (Bemba) and knew the culture of the communities were recruited to collect data. Data collectors and supervisors were trained on how to fill the questionnaire. Data quality was also controlled by close supervision, data cleaning and editing, and cross checking of the completeness of the questionnaires. The questionnaire was pre-tested in similar settings which were not part of the study area and the necessary modifications were made on some items of the questionnaire.

### Data Processing and Analysis

Data were cleaned and appended to create a new dataset containing diarrhoea, fever and pneumonia data, with a total of 1653 participants. Descriptive statistics was performed first to observe the characteristics of the variables (numbers and percentages were reported as the variables were categorical). Associations between categorical variables were assessed using chi-squared test of independency as the assumptions of a chi-squared test were satisfied. To account for complex multistage sampling design and the clustered nature of the data, vce (cluster comp) syntax in Stata version 15 command was used. Detail of sampling for the study has been described elsewhere (26). The main statistical analysis consisted of univariable and multivariable logistic regression to identify predictors of health seeking behaviours for childhood febrile illnesses in under five children in poor resource settings. An investigator led stepwise regression method was used in multiple logistic regression to select factors influencing health seeking behaviours for common childhood illnesses in under five children. The selection of variables that fit in the final multiple regression model was done by running the multiple logistic regression command with all the predictor variables and then removing those with highest *p*-values one by one from the model until only predictor variables that best predict the outcome remained in the model. Finally, the best fit model was selected based on the Akaike's Information Criterion and Bayesian Information Criterion (AIC and BIC) for the competing models. The model with smallest values for AIC and BIC compared to other models was chosen. Crude (cOR) and adjusted odds ratios (aOR) with their corresponding 95 percent confidence intervals (CI) were presented. A *p*-value of < 0.05 was considered significant. Data analysis was performed using STATA version 15 (STATA corp. college station, Taxes USA).

### Ethical Consideration

Ethical approval was granted for the HPP project by the University of Zambia, Biomedical Research Ethics Committee (UNZABREC: REF. NO. 222/2019). Authority to use the dataset was sought from the Principal Investigator for the HPP project. This study had no direct contact with the participants and hence there was no pain or discomfort and less than minimal risk was involved. The data sets did not have participants' names, but had an identification number and hence anonymity and confidentiality was guaranteed. The benefit of the study was that knowing determinants of health seeking by mothers for common childhood illnesses would help put up specific interventions aimed at improving service delivery eventually improve the management of diarrhoea, malaria and pneumonia among under 5 years' children in Zambia.

## Results

### Overall Population Description

A total of 1 653 mothers of under five children whose children had either diarrhoea, malaria, or pneumonia the past 2 weeks were interviewed. Among the children, 882/1,653 (53.4 percent) were males while 771/1,653 (46.6 percent) were females. In regards to age, 398/1,653 (24 percent) were under 1 year while 1,255/1,653 (75.9 percent) were aged between 1 year and < 5 years. Of the 1 653 children, 551 (33.6 percent) had diarrhoea, 551 (33.6 percent) had fever, and 551 (33.6 percent) had pneumonia within a fortnight before data collection. Among the mothers interviewed, 75.2 percent (1 243/1,653), [95% CI: 0.62–0.96], sought care for their child for either of the illness while 410/1,653 (24.8 percent) did not seek care for their child's illness. The married accounted for 64.6 percent (1 068/1,653) of the mothers interviewed while 585/1,653 (35.4 percent) were unmarried. Details of demographic characteristic are given in Table 1.

**Table 1:** Socio-demographic characteristics of respondents (*N* = 1 653).

**Variables**	**Frequency (*n*)**	**Percentage (%)**
**Gender of child**		
Male	882	53.4
Female	771	46.6
**Age of child in months**		
**<**12	398	24
12-59	1 255	76
**Age of mother (years)**		
<18	340	20.6
18-58	1313	79.4
**Marital status**		
Single	585	35.4
Married	1 068	64.6
**Level of education**		
Incomplete primary	876	53
Complete primary	777	47
**Ability to read**		
Yes	436	26.4
No	1 217	73.6

Chi square test was done to determine associations between categorical variables and health seeking behaviour. Table 2 shows the results between mother's socio-demographic characteristics and health seeking behaviours as determined by Pearson's chi square test of independency.

**Table 2:** Cross tabulation of the predictors of health seeking.

**Factor**	**Sought Care for Child's illness**		***P* Values**
	**No**,	**Yes**,	
	***N* = 410** **(24.8%)**	***N* = 1,243** **(75.2%)**	
Mother' age <18 year	78 (4.7%)	266 (16%)	
18–58 years	232 (20%)	977 (59.1%)	0.741^C,M^
Marital status			
Married	282 (17%)	786 (47.5%)	
Unmarried	128 (7.7%)	457 (27.6%)	0.014^C,M^
Child's age			
0–11 months	81 (4.9%)	317 (19.2%)	
12–59 months	329 (19.1%)	926 (56%)	0.158^C,M^
Sex of a Child:			
Male	211 (12.8%)	671 (40.6%)	
Female	199 (12%)	572 (34.6%)	0.698^C,M^
Education level			
Incomplete primary	384 (30.4%)	492 (40.0%)	
Complete primary and above	88 (24.0%)	298 (77.2.6%)	0.004^C,M^
Able to read:			
Yes	93 (5.6%)	343 (20.8%)	
No	317 (19.2%)	900 (54.4%)	0.359^C,M^

*Key: C, Chi-squared test of association; M, Showing that there were missing values but p-values were obtained on complete case analysis*.

Mothers with male children had higher appropriate health seeking behaviour 40.6 percent (671/1,653) than mothers with female children 34.6 percent (572/1,653). Nonetheless, There was no significant difference in health seeking between male children and female children (*p*-value = 0.741). The study further showed that children that were <12 months old 317/398 (79.60 percent) sought appropriate treatment compared to children aged more than 12 months but <60 months. There was no significant difference as well in health seeking between children aged under 1 year and those aged between 1 year and below 5 years (*p*-value = 0.158).

There was no evidence of the difference also in health seeking behaviour between those aged below 18 years and mothers aged 18 years and above (*p*-value = 0.304). Further, findings from this study revealed that those who were married sought appropriate treatment 786/1,068 (74.0 percent) compared to those that were not married. There was a significant difference in health seeking between married mothers and unmarried mothers (*p*-value = 0.014).

Mothers who completed at least primary school sought appropriate treatment 298/386 (77.2 percent) for their children compared to those that did not have primary education. There was a significant difference in health seeking between mothers who at least completed primary school compared to mothers who did not even have primary education as evident by the *p*-value (*p*-value = 0.004). Results however, showed no significant difference in health seeking between mothers who could read and those who could not read (*p*-value = 0.359).

### Predictors of Healthcare-Seeking Behaviour for Childhood Illnesses

A logistic regression was performed to examine the predictors of health seeking for febrile illnesses among mothers of under five children. Significance level was set at *p*-value of <0.05 at 95 percent confidence interval.

The results of univariate analysis, that is, crude odds ratios (cOR) in Table 3 shows that marital status of the mothers, that is, mothers who were unmarried had reduced odds of seeking appropriate health care for their sick child by 26 percent compared to mothers who were married (cOR = 0.74; 95% CI: 0.58–0.94), and mother's education level, that is, whether mother had at least completed primary school, mothers who had completed at least primary school had 1.5 times the odds of seeking appropriate health care for their child compared to mothers who had not completed primary school (cOR = 1.47; 95% CI: 1.13–1.92), were significantly associated with health care seeking behaviours. On the other hand, child's age (cOR = 1.22; 95% CI: 0.92–1.62), mother's age (cOR = 1.106; 95% CI: 0.76–1.46), mothers' ability to read (cOR = 1.13; 95% CI: 0.87–1.48) and sex of the child (cOR = 1.06:; 95% CI: 0. 0.83–1.31) had no statistically significant association with health care seeking behaviours.

**Table 3:** Logistic regression-adjusted and unadjusted.

**Factor**	**Unadjusted**			**Adjusted**		
**Health care seeking**	**Odds ratios**	**95% conf. interval**	***P*-values**	**Odds ratios**	**95% conf. interval**	***P*-values**
Mother' age						
18–58 years		Ref.				
<18 years	1.06	0.76–1.46	0.741	0.75	0.40–1.42	0.381
Child's age						
12–59 mths		Ref.				
<12 mths	1.22	0.92–1.62	0.159	1.46	0.81–2.63	0.203
Child's sex						
Females		Ref.				
Males	1.06	0.83–1.31	0.698	1.13	0.70–1.80	0.618
Marital						
Married		Ref.				
Unmarried	0.74	0.58–0.94	0.014	0.74	0.58–0.94	0.0015
Education level						
Incomplete primary		Ref.				
Complete primary and above	1.47	1.13–1.92	0.005	1.41	1.13–1.92	0.005
Able to read						
No		Ref.				
Yes	1.13	0.87–1.48	0.359	0.9	0.57–1.43	0.662

Table 4 shows the final multivariable analysis model arrived at that fits the data well. Multiple regression was done in order to control for possible confounding. An Investigator led stepwise Regression was used to arrive at the model. This implies running the multiple logistic regression command with all the predictor variables in the first stage and then removing variables with highest *p*-values one by one from the model until we remained with a model that best explained the data (parsimonious model).

**Table 4:** Multivariable analysis.

**Care seeking**	**Odds ratios**	**95% Confidence** **intervals**	***P*-values**
Child's age			
12–59 months	Ref.		
<12 months	1.2	0.91–1.60	0.196
Marital status			
Married	Ref.		
Unmarried	0.74	0.58–0.94	0.015
Sex of child			
Female	Ref.		
Male	1.06	0.84–1.33	0.623
Education level			
Incomplete primary	Ref.		
Complete primary and above	1.5	1.15–1.96	0.003

The multivariable analysis model contains four explanatory variables; child's age, sex of the child, marital status of the mother, and mother's education level as the best predictors of health seeking for childhood febrile illnesses. Although child's age and gender were not statistically significant, the variables were left in the model due to priori knowledge from other studies which consistently showed that they could be used to perfectly predict health seeking behaviours for childhood febrile illnesses.

As shown in Table 4, marital status and maternal education were significantly associated with health seeking behaviour.

The effect of marital status, that is, whether mother was currently married or unmarried was that mothers who were unmarried had reduced odds of seeking appropriate health care for their sick child by 26 percent (AOR = 0.74; 95% CI = 0.58–0.94) compared to mothers who were currently married, holding constant the effect of other predictors in the model. The other predictor was maternal education, mothers who had completed at least primary school were 1.5 times more likely to seek appropriate health care for their child (AOR =1.5; 95% CI = 1.15–1.96) compared to mothers who had not completed at least primary school holding constant the effects of other predictors in the model. On the other hand, child's age (AOR = 1.20; 95% CI: 0.91–1.60), and sex of the child (AOR = 1.06; 95% CI: 0. 0.84–1.33) had no statistically significant association with health care seeking behaviours controlling for the effect of other predictors in the model.

## Discussion

This study was conducted to determine factors associated with health seeking behaviours for common childhood illnesses among mothers of under-five years children in Luapula and Northern Provinces of Zambia. The study revealed vital findings regarding mothers' health -seeking behaviour for these common childhood illnesses (diarrhoea, malaria, and pneumonia). The finding of this study shows that three in every four mothers took their sick child aged <5 years to health facilities. This finding is consistent with other studies that determined health care seeking behaviours for common childhood illnesses in other rural settings (19, 31, 32).

Andersen-Newman Framework for Health Services Utilisation (33) indicates that utilisation of health services is influenced by various factors such as population and environmental characteristics. Our findings also have shown that various demographic characteristics such as mothers' education level and marital status were associated with health care seeking behaviours for common childhood illnesses in poor resource settings. The findings which are closer to studies done in rural Ethiopia and Nigeria that looked at socio-demographic determinants of mothers' health care seeking behaviours for febrile illnesses in under five children in rural areas (19, 24).

The current findings that three-forth (75%) of the mothers took their children with common childhood illnesses to health facilities seemingly could suggest mothers' preference and trust for seeking care from formal health facilities. This demonstrates the trust mothers may have in free health services offered in formal health facilities (24). The proportion of mothers who took their sick children to health facilities is close to the findings of other studies conducted in rural Democratic Republic of the Congo 148/290 (51 %), remote Madagascar 159/300 (53 %) and rural Nigeria 196/350 (56 %) where mothers were found to prefer designated health facilities for treatment of their sick children (19, 31, 32). The results of this study are also similar to the ones found in two other researches done in remote Uganda where it was found that under five children with common childhood illnesses were being taken to health facilities for treatment (34, 35). However, the results are contrary to the findings of some study in rural Liberia which revealed that only one third of mothers took their seek children. This discrepancy was attributed to user fees which prevented most mothers from visiting health facilities (36).

The study also showed that mothers' education level was predictive of health care seeking for their child's common childhood illness. A strong association between mothers' level of education and health seeking behaviours found in this study suggests the importance of basic education to care seeking behaviours. Various researches have revealed associations between maternal education and health care seeking behaviour, thus agreeing with findings of this study (19, 23). This finding suggests need to improve literacy rates in Zambia as a proxy to improving care-seeking behaviour. Some arguments were made by Gerald (37), that educated women easily comprehend health education and awareness messages. Hence, fostering early recognition of signs and symptoms of illness (20). This result was consistent with other studies conducted in rural Sierra-Leone and rural Ivory Coast which reported that mothers who attained with primary education or more were more likely to seek treatment from health facilities for their children (21, 36). It is also documented in some study in Uganda that the odds of seeking appropriate health care increased if mothers had completed at least primary school education (38). Hana in Yemen also reported a 6-fold likelihood of seeking medical attention among mothers with secondary education compared to mothers with less education. She therefore argued that mothers who are educated are exposed to reading materials thereby broadening their understanding of health education messages availed in various methods (17). In line with this thought, in another study that compared pregnant women's utilisation of Primary Health Care (PHC) units for Antenatal Care (ANC), it was reported that women with higher education snubbed the services at PHCs in preference for secondary care facilities. Better educated women were reported to shun the services due to perceived poor quality in preference for the same service at health facilities (39). Similarly, in other studies that looked at utilizations of Antenatal care services (ANC), maternal education were reported to be highly associated with utilizations of ANC services (40). Some researchers, however, that looked at predictors of utilizations of maternal, neonatal and child health services, question the sole independent association of maternal education on maternal, neonatal and child health services utilisation. They argue that other factors such as economic, socio-environment and husband's level of education and occupation interact to dilute the association (39, 41).

However, other studies reported otherwise. In rural Ethiopia and rural Senegal, Getahun (42) and Smith et al. (43), respectively, found that mothers' level of education was not associated with initial place of treatment. In other studies conducted in Uganda, education was not a factor in 68 percent of the caregivers' that had sought treatment from health facilities (44).

Mothers' marital status was also predictive of health seeking behaviours for common childhood illnesses in under 5 years children. In the study, married mothers were more likely to seek appropriate medical care for their sick child compared to unmarried ones. Married mothers may draw some motivation to take their sick children to health facilities from family, and husband support. Extended family support systems is common among most African cultures and traditions thus considering each community member as part of the family (16, 24). This study conquers with Kololo's (24) findings in Ethiopia who indicated that mothers who were married had higher odds of seeking appropriate medical care for their sick children perceived to have common childhood illnesses compared to mothers who were not married. Other studies conducted in Ethiopia and Sierra-Leone (16, 24), documented that the relationship between the mother and the household head was predictive of health seeking behaviours. In a related study done in India to determine factors associated with pre-natal care utilisation. The absence of would be the father to the unborn child during prenatal visits was negatively associated with pre-natal care service utilisation (40). In another study conducted in rural Nigeria that looked at barriers to utilisation of Primary Health Care Units (PHCs), it was reported that utilisation of PHC units for prenatal care among married women was higher compared to their unmarried counterparts (45).

Unlike most studies that documented an association between gender of the child and health seeking behaviours (25, 46), the study found otherwise. These studies documented an increased odds of taking a male child to the health facility compared to female children (25, 46). There was no evidence of the difference as regards to the gender of the child in the study. Likewise, there was no evidence of the difference regarding the age of the child and health seeking which was inconsistent with what was reported from Senegal, Tanzania and Bangladesh that 1 year reduction in age of an under five child resulted in increased odds of health care seeking behaviours by the mother for common childhood illnesses (25, 43, 47).

## Conclusion

The findings of the study that the majority of mothers sought health care for their children with common childhood illnesses is close to those found in other studies carried out in rural settings to determine predictors of health care seeking behaviours for common childhood illnesses. It was also established in this study that health care seeking behaviours for these common childhood illnesses in poor resource settings could be predicted by mother's level of education and marital status. Since chance finding could be ruled out, the findings of this study therefore, can be generalised to other similar settings.

As found in the current study that the majority of the mothers sought appropriate health care for common childhood illnesses, and that the level of education could be predictive of health care seeking behaviours, this therefore, offers an opportunity for health care workers to provide appropriate health care service in line with Government policy and would eventually lead to reduction in child mortality and morbidity (48). In addition, in line with government policy on health service delivery aimed at taking health care service close to the family as possible, there is need to integrate these efforts with the provision of basic education to women and girls. Education will result in increased awareness and knowledge (19, 37), thereby fostering early recognition of signs and symptoms of illness which will create demand for health services (14).

### Study Limitations and Strength

The study limitations worth noting. Firstly, the study used secondary data. As a result, there was no room to introduce new variables. In addition, the data analysed was collected in remote settings, hence it may not be generalizable to other settings. Lastly, the dataset used was relatively old, many changes would have taken place. Despite these limitations however, we strongly feel they do not significantly influence our findings because; firstly the study used the Lot Quality Assurance sampling method which was a robust rapid methodology in assessing coverages when distribution assumptions for a community during selection have been carefully carried out. This enables generalisation of results in similar settings since the study population was highly representative. Secondly, the multivariable analysis enabled us to control for any confounding demographic characteristics. Thirdly, the study also provides useful information on predictors of health seeking behaviours by mothers for febrile illnesses in resource poor settings which may inform health policy on bridging the gap to health service accessibility.

## Data Availability Statement

The data analysed in this study is subject to the following licences/restrictions: Permission to use the data sets was obtained under strict conditions that it should be used for academic purposes only, and not be made available to the public or any other organisations. Requests to access these datasets should be directed to Choolwe Jacobs, choolwe2003@yahoo.com.

## Ethics Statement

The studies involving human participants were reviewed and approved by The University of Zambia, Biomedical Ethics Committee. Written informed consent to participate in this study was provided by the participants' legal guardian/next of kin.

## Author Contributions

GA: research concept and design, preparing research proposal, data analysis and writing the manuscript. CJ: research concept and design, supervising the research process, actively involved in the data analysis and reviewing the manuscript and final editing. PM: research concept and design, supervising the research process, actively involved in the data analysis and reviewing the manuscript and final editing. All authors contributed to the article and approved the submitted version.

## Conflict of Interest

The authors declare that the research was conducted in the absence of any commercial or financial relationships that could be construed as a potential conflict of interest.
